# A novel 6-gene signature derived from tumor-infiltrating T cells and neutrophils predicts survival of bladder urothelial carcinoma

**DOI:** 10.18632/aging.203770

**Published:** 2021-12-14

**Authors:** Xuan Zou, Yong Wei, Tao Qi, Xiaping Wang, Wenren Zuo, Tongshan Wang, Wei Zhu, Xin Zhou

**Affiliations:** 1 Department of Medical Oncology, Fudan University Shanghai Cancer Center, Shanghai 200032, China; 2 Department of Oncology, Shanghai Medical College, Fudan University, Shanghai 200032, China; 3 Department of Urology, Jiangsu Province Hospital of Chinese Medicine, Affiliated Hospital of Nanjing University of Chinese Medicine, Nanjing 210029, China; 4 Department of Anesthesiology, First Affiliated Hospital of Nanjing Medical University, Nanjing 210029, China; 5 Department of Pathology, The Second Affiliated Hospital of Nanjing Medical University, Nanjing 210000, China; 6 Department of Oncology, First Affiliated Hospital of Nanjing Medical University, Nanjing 210029, China

**Keywords:** bladder urothelial carcinoma, survival, T cells, neutrophils, nomogram

## Abstract

Intratumoral immune cells were reported to be associated with prognosis of bladder urothelial carcinoma (BUC). However, the role of immune cells related genes in BUC prognosis is less well defined. In the study, we analyzed data retrieved from the Cancer Genome Atlas database and found higher neutrophils and lower T cells infiltration in BUC tumor tissues were significantly correlated with patients’ worse prognosis. Additionally, the expression levels of 164 genes were significantly correlated with T cells and neutrophils proportions. A Cox proportional-hazards model integrating 6 genes expression (EMP1, RASGRP4, HSPA1L, AHNAK, SLC1A6, and PRSS8) was identified. The 6-gene signature outperformed other clinical factors in risk prediction and was an independent prognostic factor for BUC. The findings were further conformed in three Gene Expression Omnibus datasets (n=331) and Jiangsu Province Hospital cohort (n = 46). Gene set enrichment analysis revealed that the model was highly involved in some immune-related pathways. A comprehensive nomogram combining the model and other clinical parameters was finally constructed to facilitate clinical application. In conclusion, a T cell and neutrophil-associated 6-gene prognostic model was identified for the survival prediction of BUC patients.

## INTRODUCTION

Bladder cancer (BC), a type of cancer arising from the urinary bladder tissues, is one of the most common malignancies in the urinary system. BC may develop at any age, and the risk generally increases with age. The prevalence and mortality rate of BC are higher for males than females, ranking the top ten among all malignancies [1–3]. Other well-defined risk factors of BC include tobacco use, family history of cancer, exposure of toxic chemicals, et al. [4]. The majority of BC originates from epithelial cells, and the pathological type bladder urothelial carcinoma (BUC) accounts for nearly 90% of all BC cases [5]. Treatment strategy and overall prognosis of BUC are strongly related to disease stage. For patients with early stage BUC, the general 5-year survival rate can reach more than 50%; but for patients with distant metastasis, the probability may be less than 10% [1]. At present, there is still a lack of potent or valid survival prediction models for BUC except several classical risk factors. Identification of a novel and comprehensive prognostic signature for BUC is of great significance for effectively distinguishing high-risk patients and facilitating individualized treatment to improve clinical outcomes.

Growing evidence has revealed the multifaceted role of immune-regulatory mechanisms in cancer development, progression, and recurrence, which enables the emergence of immune-related tools guiding cancer diagnosis, treatment, prevention, and prognosis [6]. The effects of immune system on cancer development are quite complex, involving the balance between tumor-promoting and tumor-suppressing immune responses [7]. Nowadays, the immune landscape of various cancer types has been gradually uncovered, giving abundant information on the distribution of immune cell populations and immune effector molecules within tumor microenvironment (TME) [8]. Tumor-infiltrating immune cells, such as T cells, macrophages, and neutrophils, are critical elements in TME and have shown close association with the clinical outcomes of various cancers [9]. However, knowledge about the immunology of BC is still insufficient to provide a foundation for clinical application. Technologies such as single-cell sequencing and high-throughput flow cytometry are still too costly to be widely used, which hampers the deep understanding about the regulatory roles of tumor immunity.

With the emergence of high-throughput data and data processing algorithms, the prediction of multi-dimensional information about cancer using single-layer data has become possible. The popular algorithms ‘CIBERSORT’ [10] and ‘ESTIMATE’ [11] are two representative examples, which enable the estimation of immune cell proportions and non-tumor components in TME based on gene expression profiles. Since the Cancer Genome Atlas (TCGA) and Gene Expression Omnibus (GEO) public databases can provide an abundant source of tumor sequencing data, applying these algorithms in multiple databases can help draw and verify the outlines of BC immunology. According to our primary analysis in TCGA database, the proportions of T cells and neutrophils are two potential prognostic factors for BUC. By selecting in T cell and neutrophil-associated genes in BUC, we suspected that a novel immune cell-correlated prognostic signature could be identified to facilitate the prediction and understanding of BUC prognosis. In this study, the Cancer Genome Atlas Urothelial Bladder Carcinoma (TCGA-BLCA) dataset was analyzed for model construction; three GEO datasets (GSE13507, GSE31684, and GSE48276) and a cohort of 46 BUC patients recruited from the Jiangsu Province Hospital (JSPH) were further analyzed for external validation. We aim to propose a potent prognostic model based on tumor immune cell proportions in BUC. Clinical- and immune-correlation analyses were also performed to explore the potential function of this novel signature.

## RESULTS

### Study design and datasets

**Figure 1:**
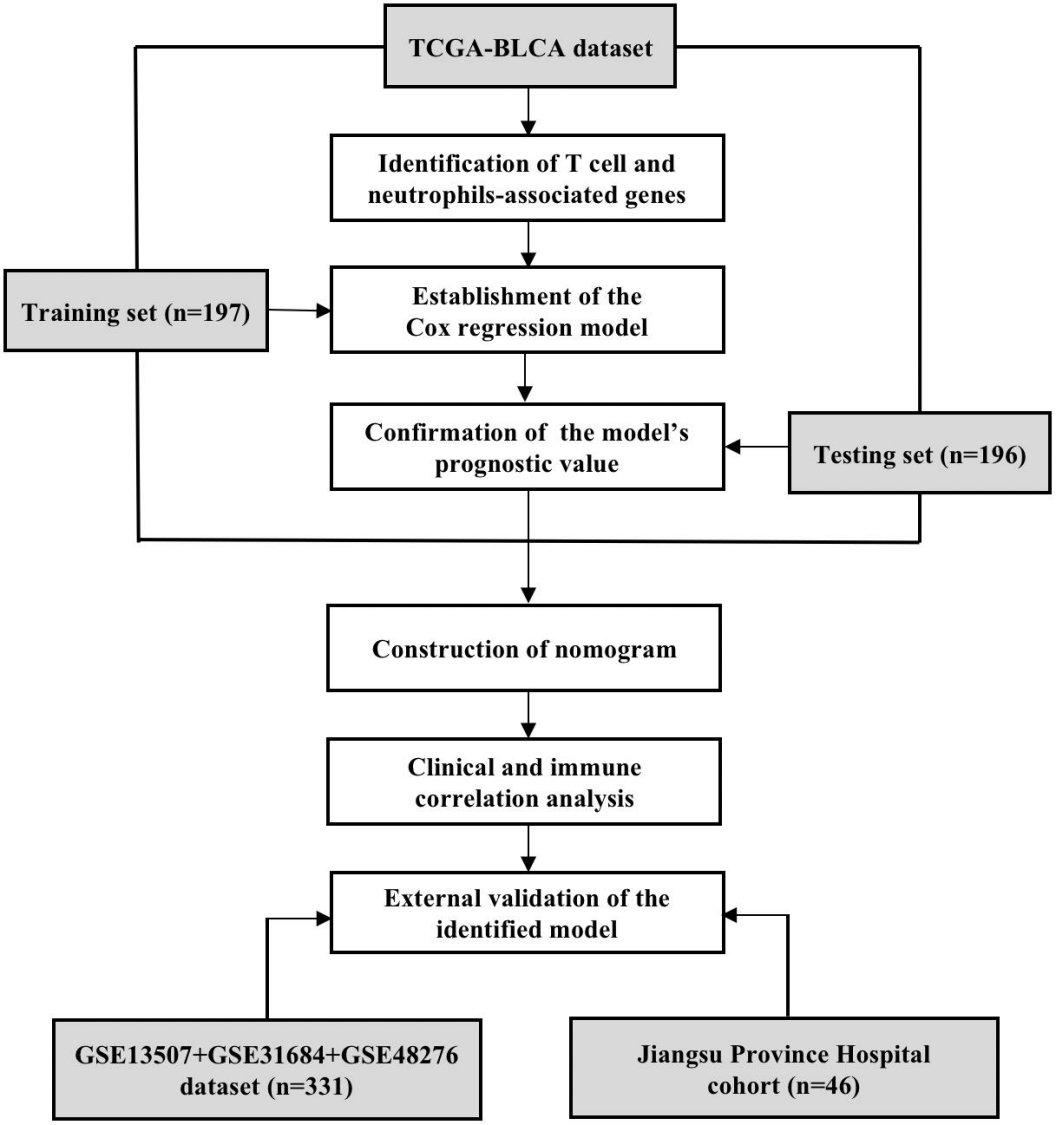
**The flow chart of study design.** The T cell and neutrophils-associated genes were identified by Spearman correlation analysis using the data of TCGA-BLCA dataset. The total 393 samples of the TCGA-BLCA dataset were then randomly divided into the training and testing sets for the construction and validation of prognostic model. The clinical- and immune-correlation of the identified model was further explored in the whole TCGA-BLCA dataset. Two independent sets, including an integrated GEO dataset and a cohort recruited from the Jiangsu Province Hospital, were further analyzed for the external validation of the model.

As the flow chart shows (Figure 1), the samples of the TCGA-BLCA dataset were randomly separated into the training (n=197) and testing (n=196) sets for the construction and verification of prognostic model, respectively. The clinicopathological features of patients in the TCGA-BLCA dataset are listed in Table 1. Chi-squared test revealed that several potential confounding factors such as age, gender, tumor-node-metastasis (TNM) stage, tumor size, lymph node metastasis, and distant metastasis were all evenly distributed in the training and testing sets with *P* > 0.05. For the external validation of the identified model, a dataset combining three GEO datasets (GSE13507, GSE31684, and GSE48276) and a cohort containing 46 BUC patients recruited from JSPH were further analyzed. The clinicopathological features of patients in the two external validation sets are shown in Table 2. The study was approved by the Institutional Ethical Committee of JSPH (ID: 2016-SRFA-148) and written informed consent had been obtained from each participant.

**Table 1:** Demographic and clinical characteristics of TCGA-BLCA patients; (P-value: the result of Chi-squared test).

	**Training set**	**Testing set**	**Total**	***P*-value**
**Number**	197	196	393	
**Age**				0.575
<70	103	108	211	
≥70	94	88	182	
**Gender**				0.262
Male	141	150	291	
Female	56	46	102	
**TNM Stage**				0.986
I	1	1	2	
II	60	63	123	
III	69	67	136	
IV	66	64	130	
Unknown	1	1	2	
**T**				0.101
T0	1	0	1	
T1	2	1	3	
T2	53	60	113	
T3	101	87	188	
T4	21	36	57	
Unknown	19	12	31	
**N**				0.353
N0	111	116	227	
N1	24	20	44	
N2	39	36	75	
N3	1	6	7	
Unknown	22	18	40	
**M**				0.135
M0	84	103	187	
M1	6	4	10	
Unknown	107	89	196	

T, tumor; N, nodes; M, metastasis.

**Table 2:** Demographic and clinical characteristics of subjects in the external validation set.

	**GSE13507**	**GSE31684**	**GSE48276**	**Total**	**JSPH cohort**
**Number**	165	93	73	331	46
**Age**					
<70	106	52	52	210	19
≥70	59	41	21	121	27
**Gender**					
Male	135	68	59	262	36
Female	30	25	14	69	10
**TNM Stage**					
I	80	10	3	93	17
II	26	17	6	49	13
III	25	42	56	123	9
IV	10	19	2	31	2
Unknown	24	5	6	35	5

JSPH, Jiangsu Province hospital.

Univariate Cox regression analysis in the TCGA-BLCA dataset revealed that the proportion of tumor-infiltrating T cells in TME was a favorable prognostic factor for BUC (hazard ratio [HR]: 0.975, 95% confidence interval [CI]: 0.963-0.988, *P* < 0.001) while neutrophils proportion was an unfavorable prognostic factor (HR: 1.082, 95% CI: 1.016-1.151, *P* = 0.013). As shown in Supplementary Table 2, a total of 164 genes whose expression levels were significantly correlated with both T cells and neutrophils proportions were defined as candidate genes for model construction (*P* < 0.05; spearman's rank correlation analysis).

### Identification of a T cell and neutrophil-associated prognostic model

**Figure 2:**
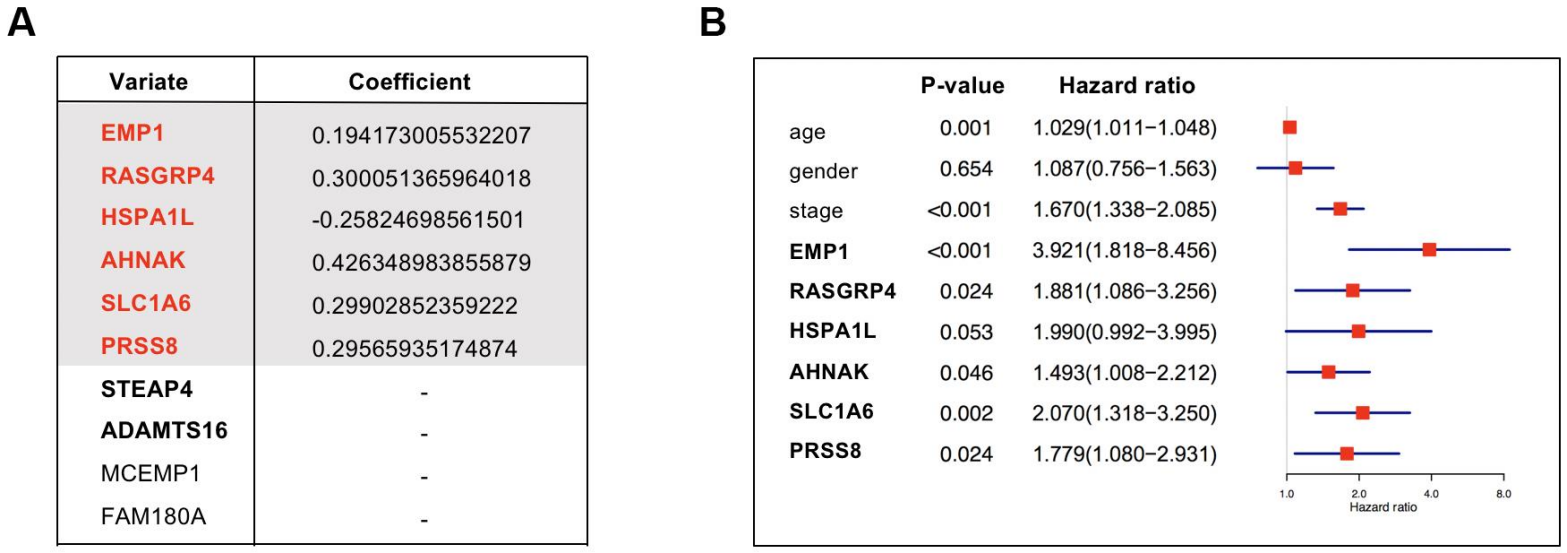
(**A**) Coefficients of the 6 covariates included in the Cox’s proportional hazards regression model. The table listed all the 10 genes with prognostic prediction value for bladder cancer according to univariate Cox regression analysis (P < 0.01) in the TCGA-BLCA dataset. Lasso regression analysis further selected the combination of 8 genes which were annotated in bold. Among the 8 genes, a model integrated 6 genes were ultimately constructed using multivariate Cox regression analysis. (**B**) The forest plot showing the prognostic value of the 6 genes. Multivariate Cox regression analysis considering gene expression levels as well as several clinicopathologic features was conducted to see if a gene was an independent prognostic factor for BUC.

Among the 164 candidate genes, 10 genes [Epithelial membrane protein 1 (EMP1), Neuroblast differentiation-associated protein (AHNAK), Solute carrier family 1 member 6 (SLC1A6), Mast cell-expressed membrane protein 1 (MCEMP1), Ras guanyl-releasing protein 4 (RASGRP4), Family with sequence similarity 180 member A (FAM180A), Serine protease 8 (PRSS8), Six transmembrane epithelial antigen of prostate 4 (STEAP4), Heat shock protein family A member 1 like (HSPA1L), and ADAM metallopeptidase with thrombospondin type 1 motif 16 (ADAMTS16)] were remarkably correlated with the prognosis of BUC patients in the TCGA training set according to the results of univariate Cox regression analysis (*P* < 0.01; Supplementary Table 3). Lasso regression analysis was further conducted to reduce redundancy (Supplementary Figure 1). Stepwise multivariate Cox regression analysis further identified a 6-gene model with the maximum prognostic value which included EMP1, RASGRP4, HSPA1L, AHNAK, SLC1A6, and PRSS8 (Figure 2A). According to the constructed Cox proportional-hazards model, risk score of a BUC patient could be calculated by the formula: 0.194173005532207 * EMP1 expression + 0.300051365964018 * RASGRP4 expression - 0.25824698561501 * HSPA1L expression + 0.426348983855879 * AHNAK expression + 0.29902852359222 * SLC1A6 expression + 0.29565935174874 * PRSS8 expression. Multivariate Cox regression analysis revealed that except HSPA1L, the other 5 genes were all independent unfavorable prognostic factors for BUC patients (HR > 1; *P* < 0.01; Figure 2B).

**Figure 3:**
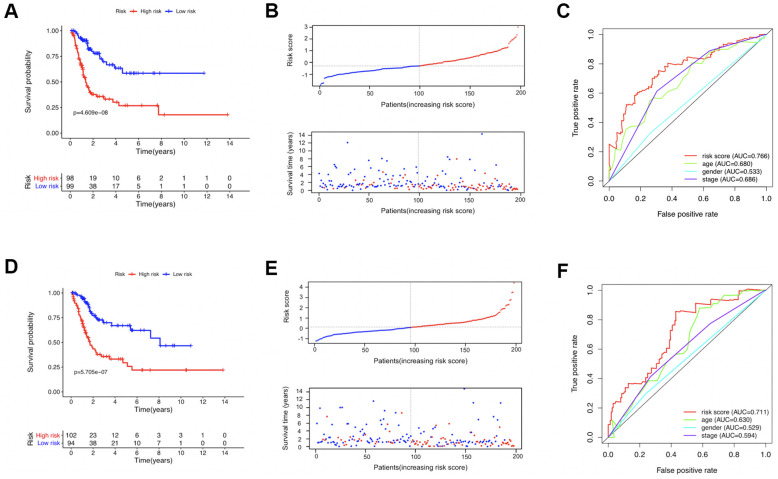
The prognostic value of the identified model in the TCGA training (**A**–**C**) and testing (**D**–**F**) sets. (**A**, **D**) Comparison of the overall survival curves between the high-risk and low-risk patients. (**B**, **E**) The distribution of survival status of patients with increasing risk scores; the red and blue dots represented being dead and alive, respectively. (**C**, **F**) The receiver operating characteristic (ROC) curves evaluating the prognostic values of several factors including the calculated risk score, age, gender, and disease stage.

In the TCGA training set, risk score of each patient was calculated. The median value of the risk scores (-0.186984284651394) was regarded as the cutoff value to distinguish high-risk and low-risk patients. Kaplan-Meier survival analysis revealed that in the training set, patients with higher risk scores had significant worse overall survival (OS) than those with lower risk scores (*P* < 0.001; Figure 3A). Generally, as the risk score increased, patients did have shorter survival time and higher mortality rate (Figure 3B). The multiple receiver operating characteristic (ROC) curves further indicated that compared with other clinical parameters, the estimated risk score [Area under the Curve (AUC) = 0.766] had higher prognostic value for the survival prediction of BUC patients (Figure 3C). In the TCGA testing set, the identified model proved to have stable prognostic performance for BUC. In this cohort, OS of the estimated high-risk patients was significantly worse than the estimated low-risk patients (Figure 3D, 3E). Moreover, risk score (AUC = 0.711) was still superior to other clinical factors for survival prediction in BUC (Figure 3F). Predictive value of the identified 6-gene model remained high when the data of the training and testing sets were combined (Supplementary Figure 2 and Table 3). In addition, expression levels of the 6 genes in tumor tissues became generally higher as risk scores increased.

**Table 3:** Multivariate cox regression analysis combining clinical characteristics and risk score.

**Variables**	**TCGA training set**		**TCGA testing set**		**Combined**	
	**HR (95% CI)**	***P*-value**	**HR (95% CI)**	***P*-value**	**HR (95% CI)**	***P*-value**
**Age**	1.026 (1.001-1.051)	0.039	1.028 (1.004-1.053)	0.022	1.030 (1.012-1.048)	0.001
**Gender**	1.419 (0.869-2.317)	0.162	0.909 (0.536-1.542)	0.723	1.075 (0.752-1.538)	0.692
**TNM Stage**	2.103 (1.463-3.023)	<0.001	1.382 (1.042-1.834)	0.025	1.610 (1.294-2.002)	<0.001
**Risk Score**	2.309 (1.728-3.085)	<0.001	1.690 (1.334-2.142)	<0.001	1.886 (1.585-2.244)	<0.001

HR, hazard ratio; CI, confidence interval.

### Construction of a comprehensive nomogram

**Figure 4:**
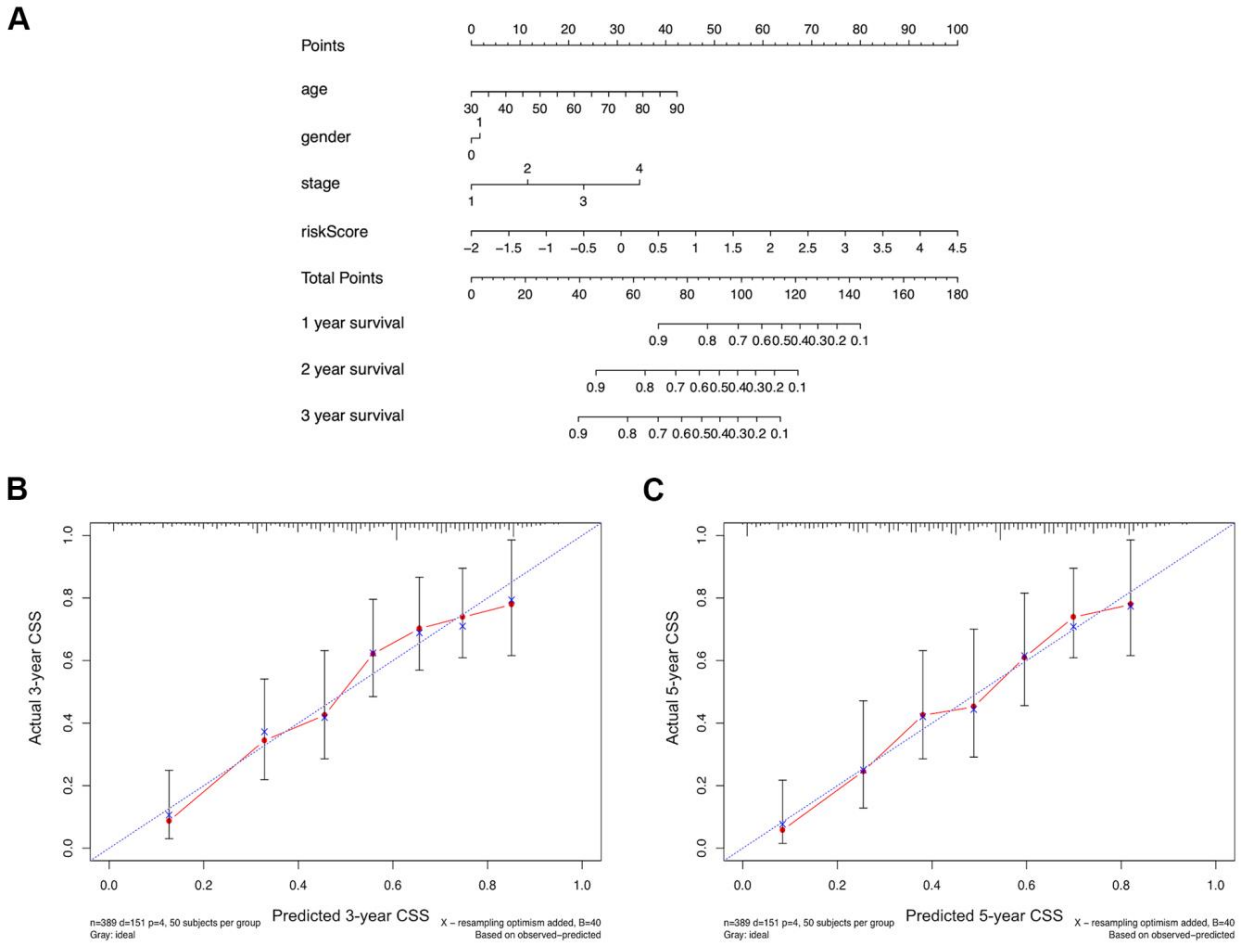
(**A**) A comprehensive nomogram integrating the estimated risk score and other clinicopathologic features for the survival prediction of bladder cancer patients using the data of TCGA-BLCA dataset. (**B**, **C**) The calibration curves comparing the predicted and actual 3-year and 5-year cancer specific survival (CSS) rates.

Based on the TCGA-BLCA dataset, a nomogram combining risk score and other clinical parameters including age, gender, and TNM stage was constructed to facilitate survival probability prediction for BUC patients (Figure 4A). Calibration curve analysis confirmed that the theoretical 3-year and 5-year cancer specific survival rates were in good agreement with the actual survival rates (Figure 4B, 4C), which demonstrated the high effectiveness of the identified signature.

### Clinical- and immune-correlation analysis

**Figure 5:**
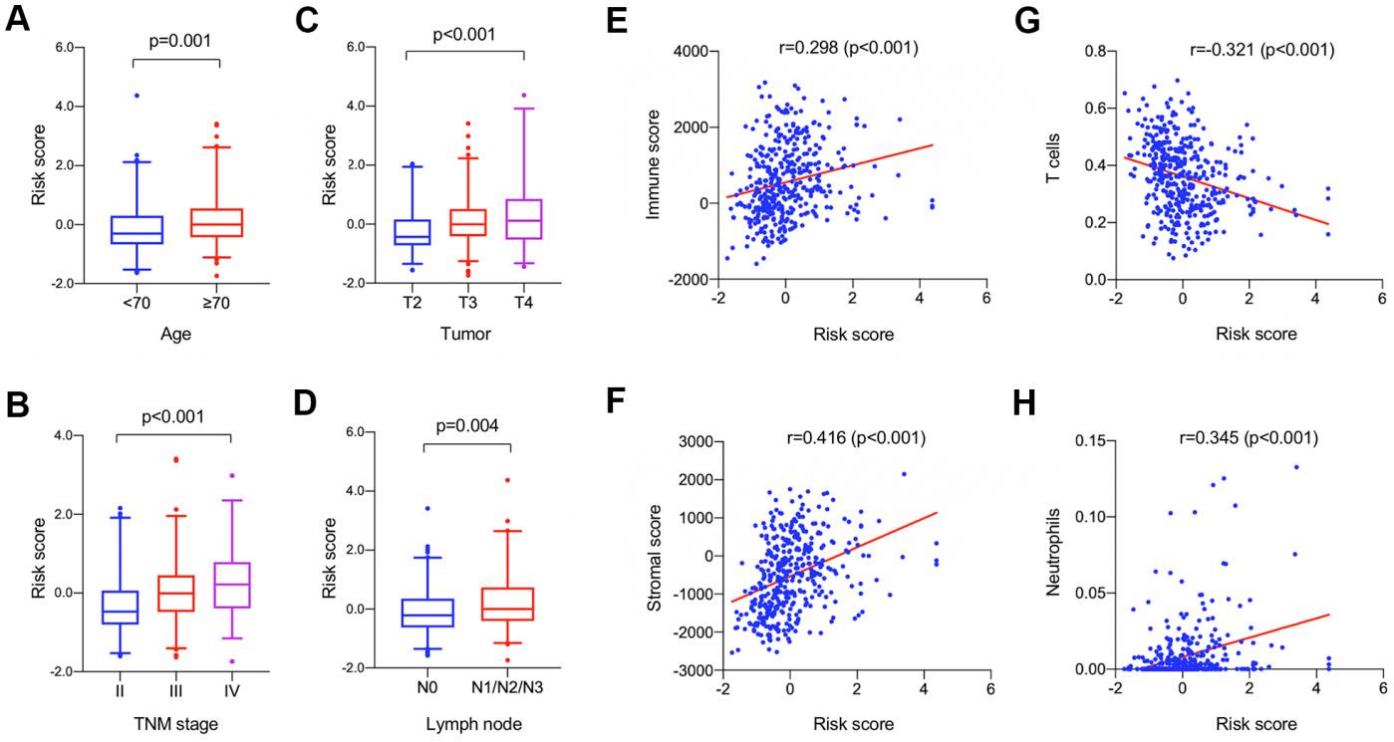
Clinical (**A**–**D**) and immune (**E**–**H**) correlation analysis of the identified model in the TCGA-BLCA dataset. (**A**–**D**) The boxplots showing the change of risk scores among patients with varied clinical characteristics including age (**A**), TNM stage (**B**), tumor size (**C**), and lymph node metastasis (**D**); horizontal lines: mean, interquartile range (Q25, Q75), and 95% confidence interval. (E-F) Spearman correlation analyses revealed that the estimated risk score was significantly correlated with the immune and stromal constituent proportion in tumor microenvironment. (**G**, **H**) The estimated score has significant negative correlation with T cell proportion and positive correlation with neutrophils proportion.

Subgroup analyses and Spearman's rank correlation analyses were performed in the TCGA-BLCA dataset to explore the association between the identified signature and clinical parameters as well as tumor immunity. As shown in Figure 5, higher risk score was associated with the condition of patient’s elder age (Figure 5A), later TNM stage (Figure 5B), higher invasion depth (Figure 5C), and more lymph node metastasis (Figure 5D). For each tumor sample, the immune score and stromal score were calculated respectively using the ESTIMATE algorithm to quantify the proportions of immune and stromal cells in TME. Estimated risk scores were in positive correlation with the contents of both immune cells (*r* = 0.298, *P* < 0.001) and stromal cells (*r* = 0.416, *P* < 0.001) in BUC tumor tissues (Figure 5E, 5F). Moreover, higher risk score was significantly correlated with less T cells (*r* = -0.321, *P* < 0.001) and more neutrophils (*r* = 0.345, *P* < 0.001) infiltration in TME (Figure 5G, 5H).

Expression levels of the 6 key genes were also compared between tumor tissues and adjacent normal tissues in TCGA-BLCA dataset. The mRNA expression levels of AHNAK, EMP1, RASGRP4, and HSPA1L were significantly down-regulated in BUC tumor tissues compared with normal tissues while SLC1A6 and PRSS8 were significantly up-regulated (*P* < 0.05; Supplementary Figure 3). Moreover, gene expression levels were further compared between BUC patients aged < 70 and ≥ 70 years. Only HSPA1L showed significantly lower expression in tumor tissues in elder BUC patients compared with the younger counterparts (*P* = 0.021; Supplementary Figure 4); there were no significant expressing differences for the other 5 genes. The typical immunostaining graphs of these genes in BUC tumor tissues were provided by the human protein atlas (HPA) database (Supplementary Figure 5).

### Functional exploration by gene set enrichment analysis (GSEA)

**Figure 6:**
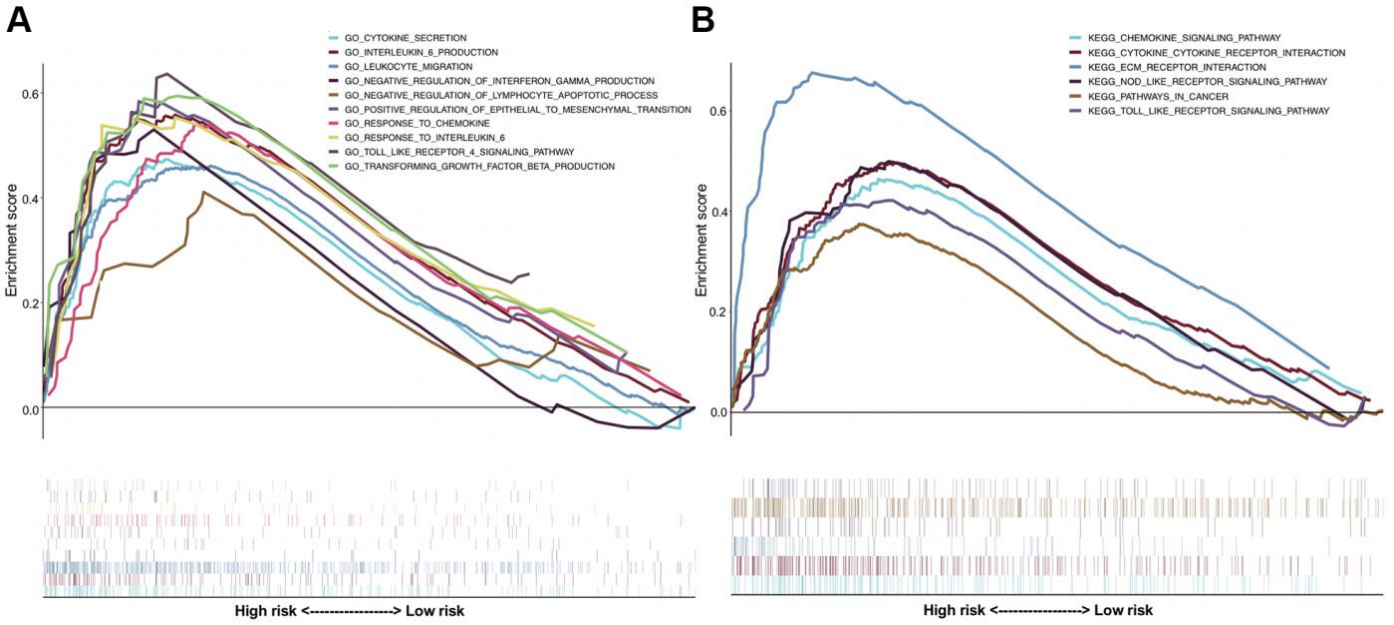
**Gene set enrichment analysis (GSEA) comparing the significantly differential pathways involved by the high-risk and low-risk patients.** (**A**) The results of Gene Ontology (GO) biological processes analysis; (**B**) the results of Kyoto Encyclopedia of Genes and Genomes (KEGG) pathway analysis.

In the TCGA-BLCA dataset, the differential pathways and biological processes enriched in the high-risk and low-risk groups were listed in Supplementary Table 4.1, 4.2 (false discovery rate [FDR] < 0.05). A number of immune-related pathways (*i.e.*, ‘cytokine secretion’, ‘interleukin-6 production’, ‘leukocyte migration’, ‘cytokine-cytokine receptor interaction’) were identified. Figure 6 lists some of the significant immune-related pathways involved in the high-risk groups compared with the low-risk groups. The results further demonstrated the close relationship between the identified signature and the immune status of BUC.

### External validation in GEO datasets

**Figure 7:**
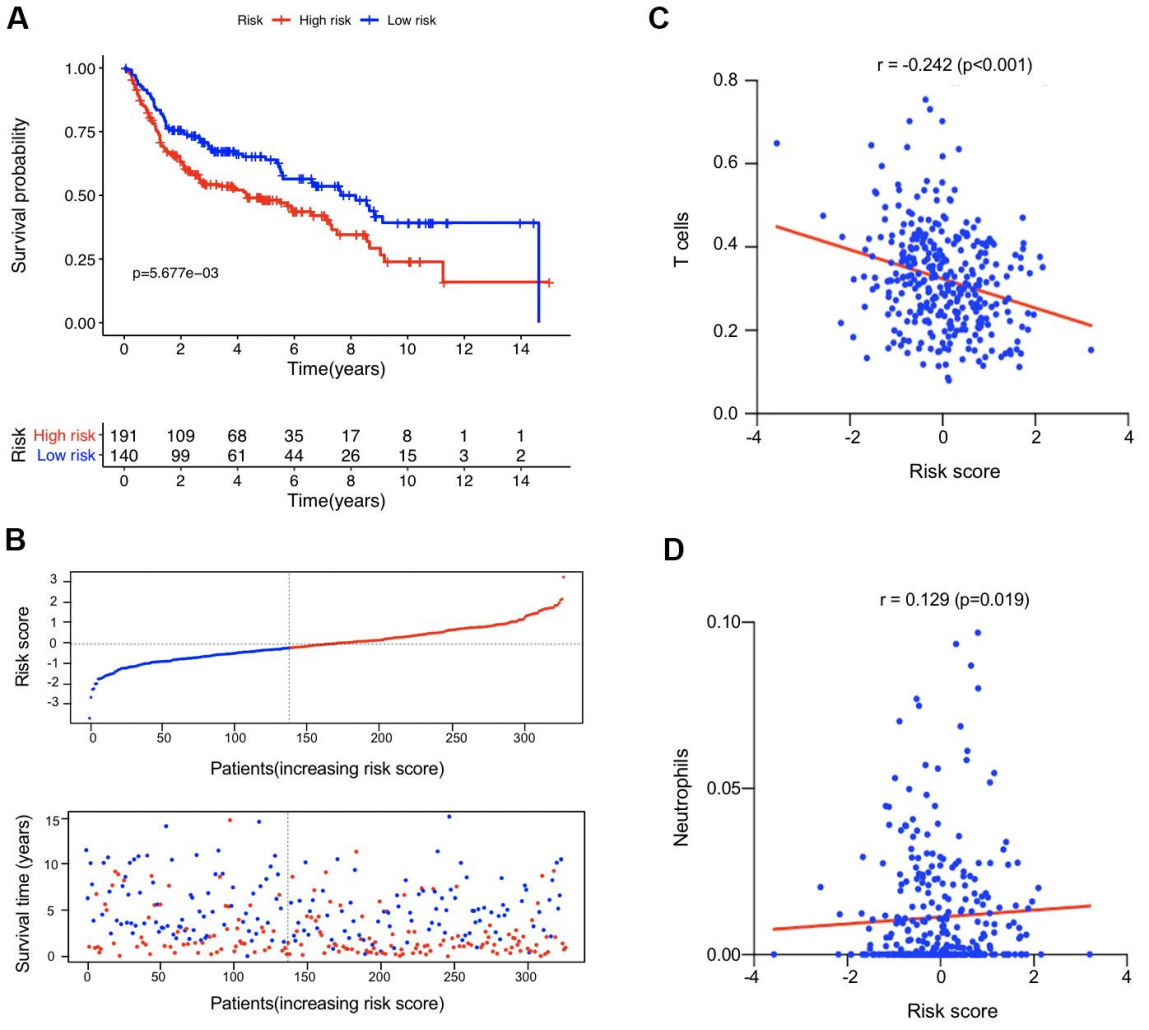
**External validation in the combined GSE13507, GSE31684, and GSE48276 datasets (n = 331).** (**A**) Kaplan-Meier survival analysis comparing the overall survival status between the high-risk and low-risk patients. (**B**) Distribution plot of the risk score and survival status of each patients. (**C**, **D**) The correlation between the estimated risk score and the proportion of T cells and neutrophils in tumor microenvironment.

In the combined GSE13507, GSE31684, and GSE48276 dataset, risk scores of the 331 BUC patients were calculated using the same formula, and patients were divided into high- and low-risk groups based on the established cutoff value. OS of the predicted high-risk group was significant worse than that of the low-risk group (Figure 7A, 7B). Consistent with the TCGA-BLCA dataset, patients’ risk score had significantly negative correlation with T cells proportions in TME; while for neutrophils, the relationship was reversely positive (Figure 7C, 7D). It was further confirmed that tumor-infiltrating neutrophils and T cells might be unfavorable and favorable prognostic factors, respectively, on the long-term survival of BUC patients. For BUC, the identified T cell and neutrophil-associated 6-gene signature had potent prognostic value.

### Prognostic role of the 6 key genes in JSPH cohort

**Figure 8:**
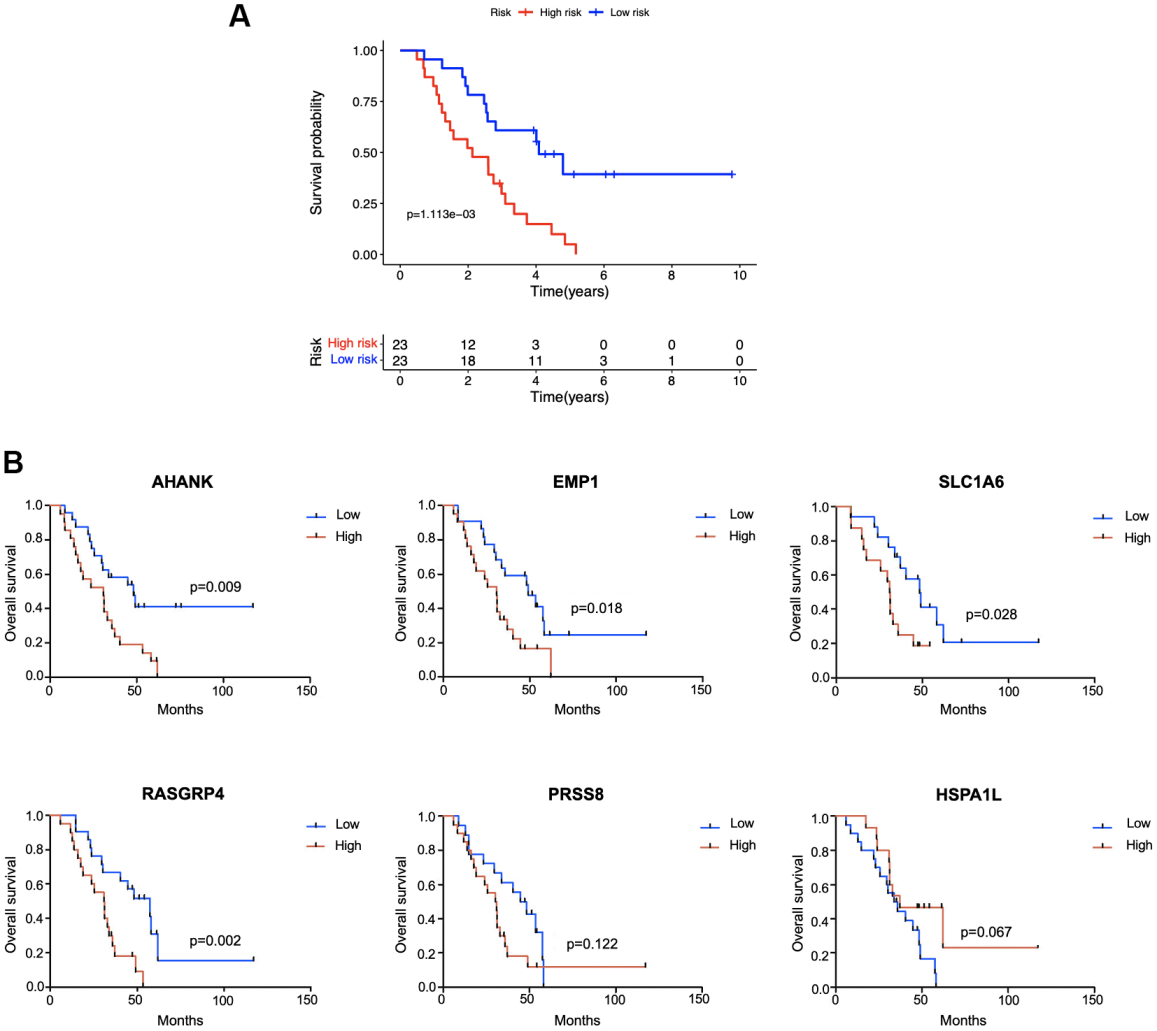
**External validation in the Jiangsu Province Hospital cohort (n = 46).** (**A**) Kaplan-Meier survival analysis comparing the prognosis between high-risk and low-risk patients. (**B**) According to the median expression level of each gene in tumor tissue samples, patients were divided into high- and low-expression groups and the survival status of the two subgroups was compared by Kaplan-Meier survival analysis.

Expression levels of the 6 genes in 46 BUC tumor tissues were analyzed by quantitative reverse transcription polymerase chain reaction (qRT-PCR) in the JSPH cohort. After z-score normalization of gene expression levels, risk scores of BUC patients in the JSPH cohort were calculated according to the identified model. As shown in Figure 8A, in the JSPH cohort, patients with higher risk scores had significant worse OS than low-risk patients (*P* < 0.05; Kaplan-Meier survival analysis). For each of the 6 genes, BUC patients were divided into high- and low-expression groups according to median expression values, and the OS rates of the two subgroups were further compared by Kaplan-Meier survival analysis. As shown in Figure 8B, patients with higher AHANK, EMP1, SLC1A6, and RASGRP4 expression had significant worse OS than the corresponding low-expression group with *P* < 0.05.

## DISCUSSION

In this study, the proportions of tumor-infiltrating immune cells in BUC were calculated using the ‘CIBERSORT’ algorithm based on the data of TCGA-BLCA project. Cox regression analysis revealed that the numbers of intratumoral T cells and neutrophils were favorable and unfavorable prognostic factors for BUC, respectively. Therefore, we proposed a novel prognostic model integrating T cell and neutrophil-associated genes to help stratify the risk of BUC patients.

T cells are composed of different subtypes with complicated phenotypes and functions, and tumor-infiltrating T cells play an extremely important role in the immune response system. The prognostic value of T cell subtypes infiltration in BUC has been revealed by several previous studies, but the exact function of a specific subtype can be inconsistent between studies. For example, the multiple states of intratumoral CD4+ T cells and regulatory T cells were discovered by David et al. [12]*.* Some studies showed that higher infiltration of CD8+ T cells was associated with better clinical outcomes in BC [13–15], but some studies came to the opposite conclusion [16]. In this study, we found that the total T-cell count infiltrating in tumor tissues instead of a specific cell sub-population was significantly correlated with the better prognosis of BUC patients. Neutrophils, key members of white blood cells, are an essential part of the innate immune response [17]. In BC, intratumoral neutrophils were found to show tumorigenic activity, and the proportion of neutrophils in blood circulation and tumor tissues was found much higher than the normal controls [8, 18]. A lot of cohort studies have demonstrated that higher neutrophil-to-lymphocyte ratio could predict the worse prognosis of BC patients, confirming the tumor-promoting role of neutrophils in BC together with the present study [19–21].

Appropriate disease biomarkers should be simple, stable, and cheap. Though the counts of intratumoral T cells and neutrophils could act as prognostic markers for BC, detecting technology can be quite complicated and expensive [22]. Instead, comprehensive biomarkers integrating expression levels of several genes can be more informative and easier to be quantified. In this study, a total of 164 genes significantly correlated with both T cells and neutrophils proportions were screened out as the candidate genes for model construction.

In this study, the T cell and neutrophil-associated Cox proportional-hazards model was constructed and verified based on the data of the TCGA-BLCA dataset, one of the biggest datasets recording the most comprehensive information of BUC samples. Stepwise multivariate Cox regression analysis identified a 6-gene signature integrating the expression levels of EMP1, RASGRP4, HSPA1L, AHNAK, SLC1A6, and PRSS8 to predict the clinical outcomes of BUC patients. The risk score of a patient could be calculated using the specific coefficients of these 6 genes. Optimal cutoff value was also specified to divide patients into high-risk and low-risk groups. Survival analysis revealed that the estimated high-risk patients had a significant worse prognosis than the low-risk patients. The model proved to be a prognostic factor for BUC independent of other potential risk factors including TNM stage, gender, and age. Moreover, the model was also superior to other clinical parameters in predictive performance with higher AUC values. The prognostic value of the identified signature was then successfully verified in an external validation set combining three GEO datasets (GSE13507, GSE31684, and GSE48276) and the JSPH cohort. To improve the clinical utility of the biomarker, a nomogram combining risk score and other clinical parameters was finally constructed to predict the risk probability of a new case. The survival rate estimated by the nomogram was in the high consistence with the actual rate.

The clinical- and immune-correlation of the identified signature was further analyzed. Briefly, the theoretical risk score was higher in BUC patients with advanced diseases, which was consistent with the actual state. Unsurprisingly, the identified marker also showed close correlation with intratumoral T cell and neutrophil proportions, and the relationship was further confirmed in the combined GEO dataset. GSEA revealed that immune-related pathways were highly involved in this risk score system, demonstrating the critical role of immune regulation in the development and progress of BUC. These results altogether showed the clinical utility and functional implications of the identified 6-gene Cox proportional-hazards model.

The prognostic roles of the 6 key genes were further verified in an additional JSPH cohort containing 46 BUC patients. Multivariate Cox regression analysis in the TCGA-BLCA dataset revealed that the expression levels of EMP1, RASGRP4, AHNAK, SLC1A6, and PRSS8 in tumor tissues were independent predictors for the unfavorable prognosis of BUC patients. In the JSPH cohort, BUC patients with higher EMP1, RASGRP4, AHNAK and SLC1A6 expression also had significant worse OS. EMP1 has been found to be a tumor promoter gene in pediatric leukemia, non-small cell lung cancer, and glioma [23–25]. As a target of c-myc, EMP1 plays an important role in promoting cell proliferation [26]. However, contradictory findings existed among previous studies as for the function of EMP1 in BC, which might be caused by differences in study populations or methods [27–29]. The correlation between EMP1 and immune cell (*i.e.*, neutrophil, regulatory T cell) infiltration in BC tumor tissues has also been discovered by previous studies, but the exact role of EMP1 in BC tumor immunity is still unclear [28]. The neuroblast differentiation-associated protein AHNAK can promote tumor metastasis by inducing TGFβ-mediated epithelial-mesenchymal transition [30]. RASGRP4 is a type of Ras activator which is involved in many key biological pathways [31]. HSPA1L is one of the most recognized cancer-related chaperones, modulating multiple biological processes in various cancers [32]. PRSS8 was typically regarded as a tumor suppressor in some cancer types including hepatocellular carcinoma and colorectal cancer [33, 34]. The association between the 6 key genes and BC tumor biology or immunity has been rarely studied and still requires deep mechanism research.

The main purpose of the present study was to establish a T cell and neutrophil-associated prognostic model for BUC. Through multiple verification in the training, testing, and external validation sets, a 6-gene model with high prognostic value was identified. However, some limitations of this study cannot be ignored. For example, although the accuracy of ‘CIBERSORT’ algorithm had been strictly validated by the developer, the proportion of intratumoral immune cell used in this study was still a theoretical value. The exact regulating role of these key genes in the immune system of BC is also largely unclear. Moreover, prospective study is still required to testify the clinical utility of the identified biomarker. Despite these objective limitations, the identified 6-gene model has shown great potential in predicting the prognosis of BC patients, which might help the early discrimination of high-risk BC cases and promote more individualized treatment for better clinical outcomes in the future.

## MATERIALS AND METHODS

### Data source and processing methods

The survival time and clinicopathologic information of all the patients with primary BUC was downloaded from TCGA-BLCA project. Subjects with follow-up period less than 30 days were excluded. All the 393 samples in the TCGA-BLCA dataset were randomly divided into the training and testing sets following the principle that clinicopathologic features including age, gender, and TNM stage were evenly distributed in the two groups to avoid potential confounding effects. Tumor transcriptome profiling data including raw counts and Fragments Per Kilobase of transcript per Million (FPKM)-normalized RNA-seq data were obtained using the gdc-client tool (https://portal.gdc.cancer.gov/). The human reference genome assembly GRCh38 was used to annotate protein-encoding genes. The transcriptome expression profiles of 18,321 protein-coding genes were normalized using ‘limma’ R package [35] and integrated into a matrix for future analysis.

GSE13507, GSE31684, and GSE48276 datasets with complete gene expression information and overall survival time of BUC patients were downloaded from GEO (https://www.ncbi.nlm.nih.gov/) for external validation. The corresponding platform files (GPL6102, GPL570, and GPL14951) of each array were further downloaded for the annotation of gene symbols. In total, 331 BUC patients from the three datasets with follow-up time more than 1 month were combined to enlarge the sample size. The ‘sva’ [36] and ‘limma’ [35] R packages were used to remove batch effect and normalize expressing data, respectively. The gene expression profiles and survival information of the three datasets were thus combined into one matrix for further analysis. The z-score normalization method was used to compare the different data types of RNA-seq and microarray.

### Proportion calculation of tumor-infiltrating immune cells

The ‘CIBERSORT’ algorithm was applied to calculate the proportions of tumor-infiltrating leukocytes in BUC tumor tissues [10]. According to the developers’ instruction, the ‘CIBERSORT’ analytical tool (https://cibersort.stanford.edu/) could accurately quantify cell fractions by the deconvolution of bulk tumor gene expression profiles rather than depend on traditional cell enumeration methods such as flow cytometry and immunohistochemistry. The signature file ‘LM22’ consisting of characteristic genes that specifically distinguish 22 mature immune cells was downloaded for reference. The normalized gene expression matrix of the TCGA-BLCA dataset and the combined GEO dataset was separately uploaded to the online application to generate the corresponding leukocyte proportion matrix. For each sample, a deconvolution *P*-value was calculated to quantify deconvolution confidence. Only samples with *P*-value < 0.05 were considered for further analysis. In addition, the fraction of immune and stromal cells in tumor tissues was further estimated using the ‘ESTIMATE’ algorithm following the author’s instruction [11]. The calculated ‘Immune score’ and ‘Stromal score’ represented the relative proportions of tumor-infiltrating immune and stromal cells, respectively.

### Selection of candidate genes

The proportion of overall tumor-infiltrating T cells in each BUC sample was calculated by adding together the percentages of ‘CD8^+^ T cells’, ‘naïve CD4^+^ T cells’, ‘resting memory CD4^+^ T cells’, ‘activated memory CD4^+^ T cells’, ‘follicular helper T cells’, ‘regulatory T cells (Tregs)’, and ‘gamma delta T cells’ according to the results of CIBERSORT deconvolution. Univariate Cox regression analysis indicated that the proportions of T cells and neutrophils in BUC tumor tissues were significantly associated with the OS of patients in the TCGA-BLCA dataset (*P* < 0.05), which gave rise to the possibility of identifying a T cell and neutrophil-associated prognostic signature for BUC. Therefore, candidate genes for model construction were screened out following the three main criteria: (i) gene expression levels were significantly correlated with the proportions of T cells and neutrophils in tumor tissues (*P* < 0.05, spearman's rank correlation analysis); (ii) the average read count of the gene was more than 10 to ensure adequate abundance for detection; (iii) the gene was significantly associated with the prognosis of BUC patients (*P* < 0.01, univariate Cox regression analysis).

### Construction of a cox proportional-hazards model and nomogram

The TCGA-BLCA training set was analyzed for the construction of prognostic model. In order to minimize overfitting, Lasso regression analysis was firstly performed for variable selection using the ‘glmnet’ R package [37]. Stepwise multivariable regression analysis was then conducted to determine the covariates in the Cox proportional-hazards model. The risk score of each patient in the training, testing, and external validation sets was calculated separately according to the coefficients estimated by the model. The median of the risk scores in the training set was regarded as the cutoff value to determine high-risk and low-risk patients. In addition, a nomogram combining the estimated risk score and several clinical parameters was further constructed in the TCGA-BLCA dataset to facilitate prognostic prediction of BUC patients.

### Functional enrichment analysis

GSEA was performed to explore the underlying function of the identified signature. The transcriptome profiling data was compared between high-risk and low-risk patients in the TCGA-BLCA dataset. The classical gene sets of Kyoto Encyclopedia of Genes and Genomes pathways and Gene Ontology project were analyzed to decipher the phenotypic differences between the two groups. For each analytical process, enrichment score (ES) and significance of ES were estimated, and normalized enrichment score and FDR were further calculated to examine the results of functional enrichment analyses. An FDR cutoff value of 5% was considered in this test.

### QRT-PCR verification in tissue samples

Paraffin-embedded tissue specimens were collected from a total of 46 BUC patients who underwent operations. With the approval of the Institutional Ethical Committee (ID: 2016-SRFA-148), these patients were recruited from JSPH during 2011 and 2017 according to agreement principle. Patients were actively followed for survival information. Total RNA was extracted from tissue specimens using RecoverAll™ Total Nucleic Acid Isolation Kit (Ambion, Austin, TX, USA). Reverse transcription and qRT-PCR reactions were performed using PrimeScript™ RT reagent Kit (Takara, Kyoto, Japan) and SYBR^®^ Premix Ex Taq II (Tli RNaseH Plus) (Takara, Kyoto, Japan) following the manufacturer’s protocols, respectively. The sequences of PCR premiers used in this study were listed in Supplementary Table 1. GAPDH was considered as a reference gene for normalization, and the 2^-∆∆Ct^ method was used to analyze the relative expression of target genes [38]. Kaplan-Meier survival analysis was conducted to assess the association between gene expression levels and BUC prognosis.

### Statistical methods

Chi-squared test was performed to determine whether the distribution of clinicopathologic characteristics was balanced between the TCGA training and testing sets. The clinical outcomes between patients with high and low risk scores were compared by Kaplan-Meier survival analysis. ROC curve analyses were conducted to assess the discriminating capability of the identified markers and make comparison with other clinicopathologic parameters. Calibration curve analysis was further considered to measure the predictive performance of the model. Multivariate Cox regression analysis was conducted to identify independent prognostic factors. Risk scores were compared between different subgroups using Mann-Whitney U test or Kruskal-Wallis H test. The correlation between risk score and immune cell fraction was assessed by Spearman's rank correlation analysis. R3.6.5 (https://www.r-project.org) and SPSS 25.0 (SPSS Inc., Chicago, IL, USA) software were applied for data analysis and graphing. A two-tailed *P*-value less than 0.05 was statistically significant.

### Data availability

The data used to support the findings of this study are included within the article.

## Supplementary Material

Supplementary Figures

Supplementary Table 1

Supplementary Table 2

Supplementary Table 3

Supplementary Table 4.1

Supplementary Table 4.2
