# Supplementary material for: A novel 6-gene signature derived from tumor-infiltrating T cells and neutrophils predicts survival of bladder urothelial carcinoma
Source: Aging (Albany NY). 2021 Dec 14;13(23):25496–517. doi: 10.18632/aging.203770 (PMC8714163; doi:10.18632/aging.203770)
Supplement: Supplementary Figures [file aging-13-203770-s001.pdf]

SUPPLEMENTARY FIGURES

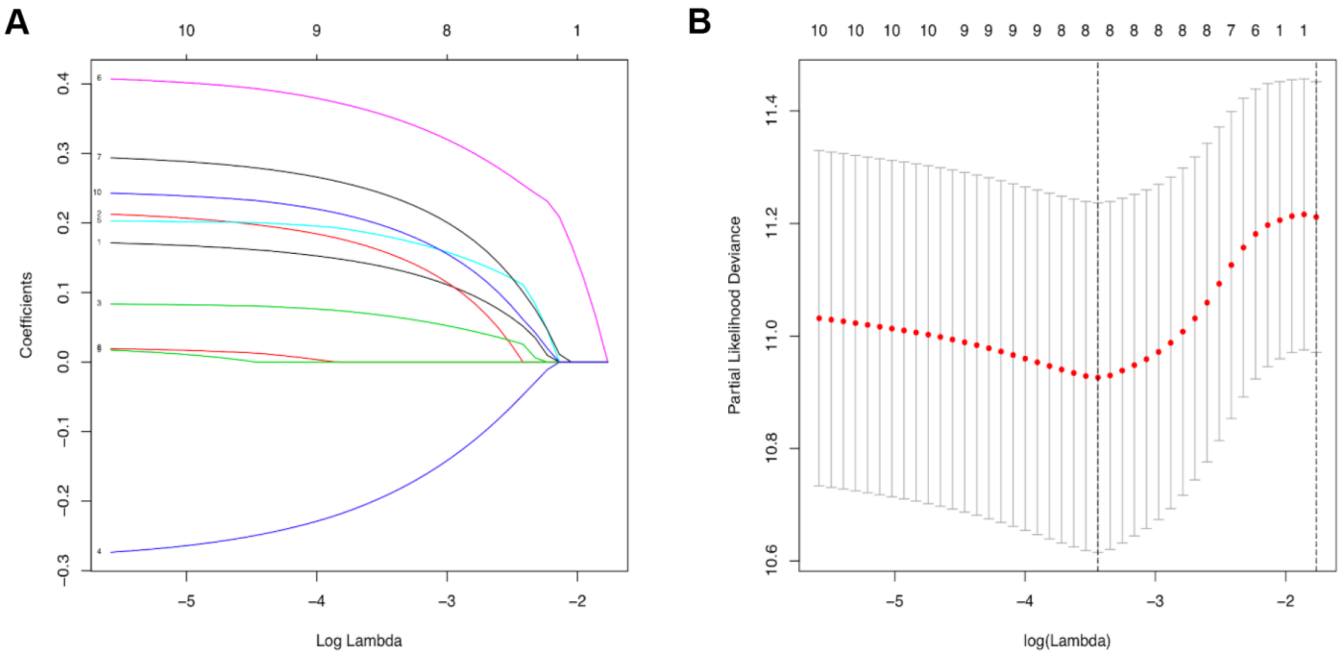

Supplementary Figure 1. Lasso regression analysis screened out 8 genes without redundancy. Lasso coefficient profiles of the T cell and neutrophil-associated genes (**A**). A coefficient profile plot against the log (lambda) sequence was generated to select the optimal parameter (lambda) in the Lasso model (**B**).

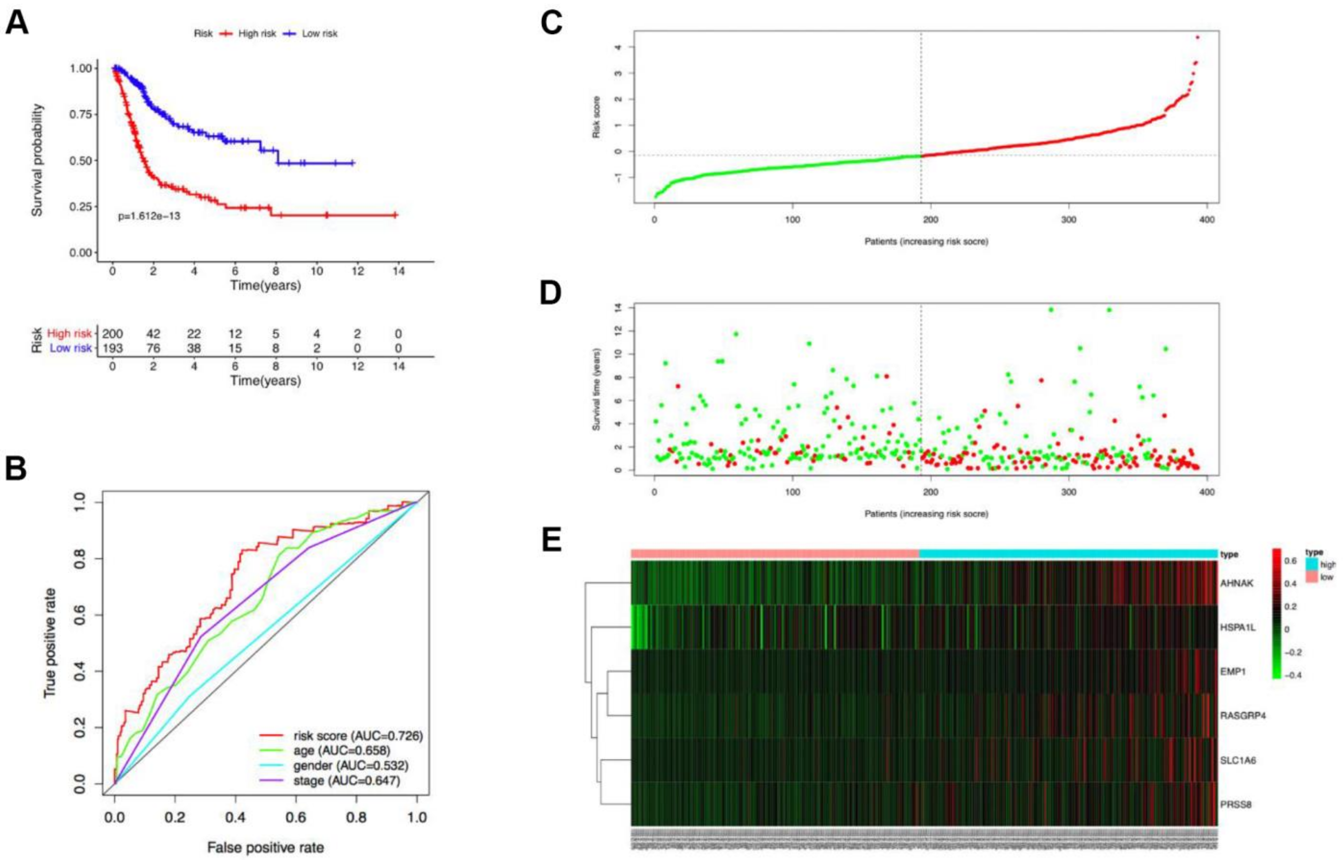

Supplementary Figure 2. The prognostic performance of the identified model in the whole TCGA-BLCA dataset. (**A**) Comparison of the overall survival between the estimated high-risk and low-risk patients. (**B**) The survival receiver operating characteristic (ROC) curves comparing prognostic value of the model and other clinical factors. (**C**–**E**) The change of patients' survival status (red dot represents dead case) and gene expression levels when risk score increased.

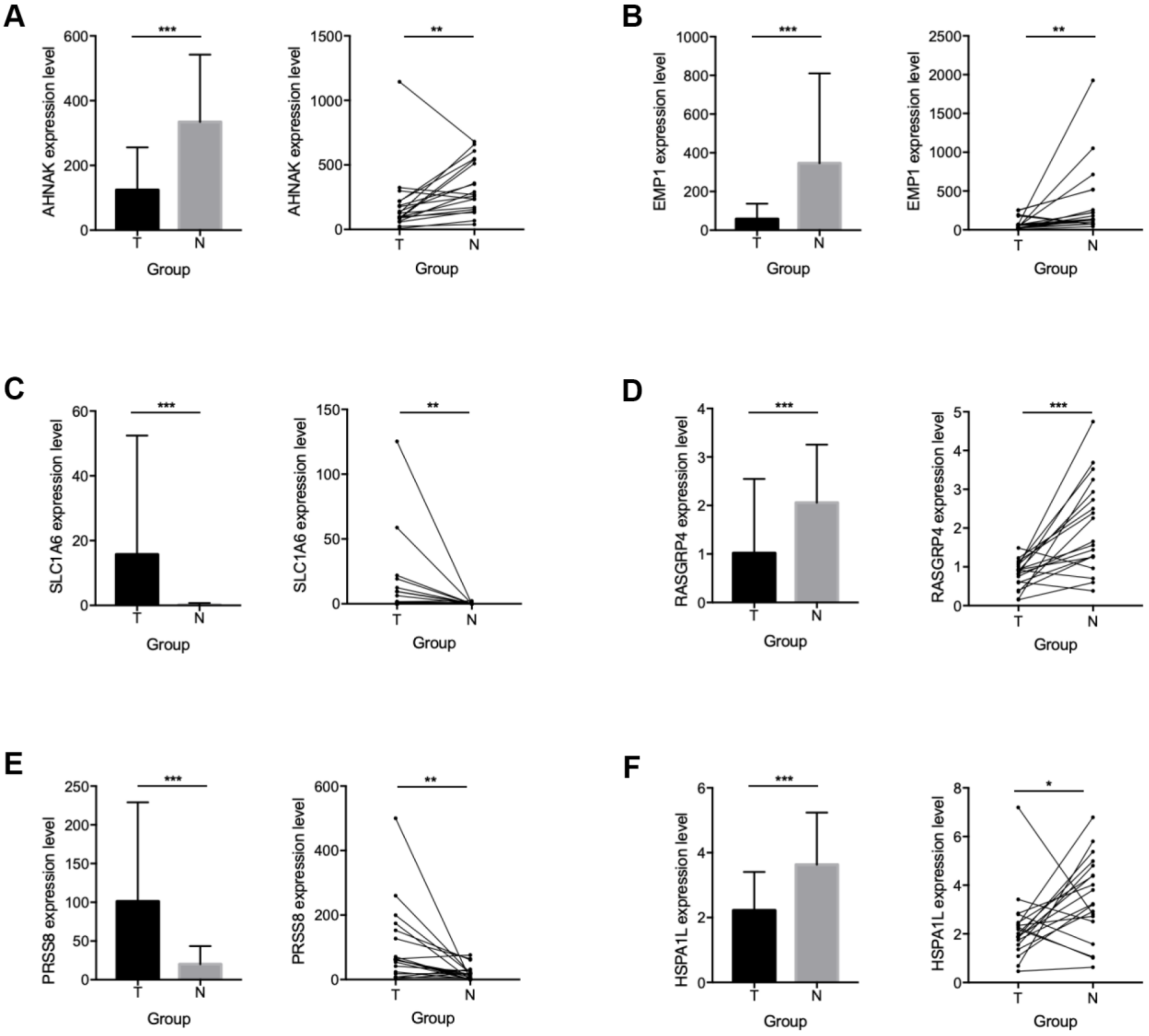

Supplementary Figure 3. Expression levels of the 6 key genes in BUC tumor tissues and adjacent normal tissues in the TCGA-BLCA dataset. (**A**) AHNAK; (**B**) EMP1; (**C**) SLC1A6; (**D**) RASGRP4; (**E**) PRSS8; (**F**) HSPA1L. Unpaired (left) and paired (right) nonparametric tests were performed for comparison. *** $P < 0.001$, ** $P < 0.01$, * $P < 0.05$.

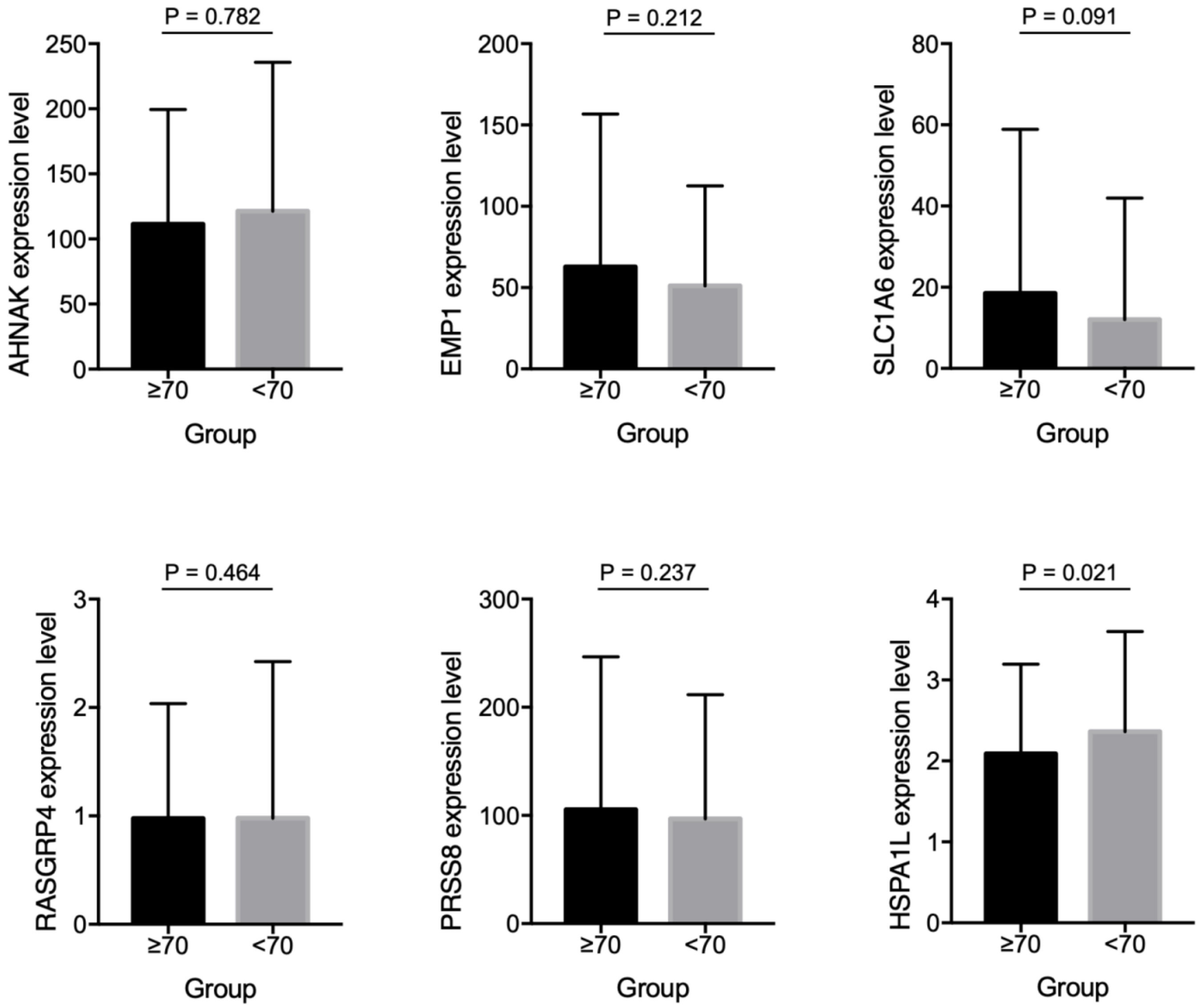

Supplementary Figure 4. Expression levels of the 6 key genes in different age groups. Mann-Whitney test was performed.

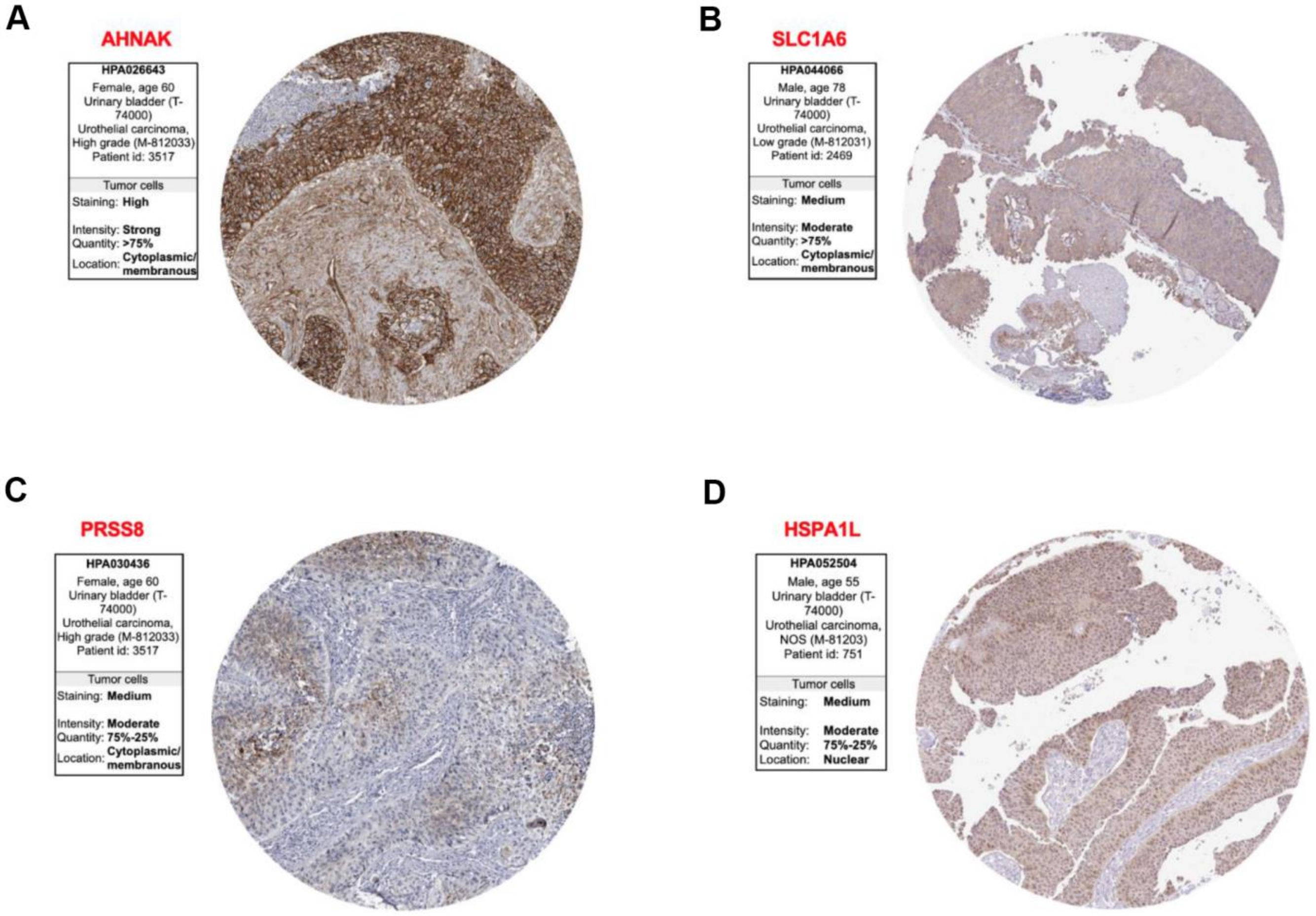

Supplementary Figure 5. Typical immunostaining graphs of the identified key genes in BUC tumor tissues provided in the human protein atlas database. (**A**) AHNAK; (**B**) SLC1A6; (**C**) PRSS8; (**D**) HSPA1L.
