# Supplementary material for: A novel 6-gene signature derived from tumor-infiltrating T cells and neutrophils predicts survival of bladder urothelial carcinoma
Source: Aging (Albany NY). 2021 Dec 14;13(23):25496–517. doi: 10.18632/aging.203770 (PMC8714163; doi:10.18632/aging.203770)
Supplement: Supplementary Table 1 [file aging-13-203770-s002.pdf]

Supplementary Table 1. The sequences of primers used in this study.

Gene	Forward premier (5' to 3')	Reverse premier (5' to 3')
**EMP1**	GTGTTCCAGCTCTTCACCATGG	GGAATAGCCGTGGTGATACTGC
**RASGRP4**	GCACAGGTGCTGGACAAGTTCA	GTCCTTGAGTCTGGAGATGGCA
**HSPA1L**	AGCGGCTGCTTCAGGACTACTT	CCATCAGGATGGCTGCTTGTAC
**AHNAK**	CGTGAAGTCTTCAGCTCCTGCA	GAGGTCTCCTTCCACTCCATCT
**SLC1A6**	AACACAAGGGCAGAGTCCTCAG	CCAGCAATCAGGAACAGGATGC
**PRSS8**	CAGCATCACCTATGAAGGCGTC	TCCTCGGAGTAGGAGTCTAGCT
**GAPDH**	GTCTCCTCTGACTTCAACAGCG	ACCACCCTGTTGCTGTAGCCAA
